# ACSL3 is an unfavorable prognostic marker in cholangiocarcinoma patients and confers ferroptosis resistance in cholangiocarcinoma cells

**DOI:** 10.1038/s41698-024-00783-8

**Published:** 2024-12-20

**Authors:** Apiwit Sae-Fung, Nawaporn Vinayavekhin, Bengt Fadeel, Siriporn Jitkaew

**Affiliations:** 1 Graduate Program in Clinical Biochemistry and Molecular Medicine, Department of Clinical Chemistry, Faculty of Allied Health Sciences, Chulalongkorn University, Bangkok, Thailand; 2 Division of Molecular Toxicology, Institute of Environmental Medicine, Karolinska Institutet, Stockholm, Sweden; 3 Center of Excellence in Natural Products Chemistry, Department of Chemistry, Faculty of Science, Chulalongkorn University, Bangkok, Thailand; 4 Center of Excellence for Cancer and Inflammation, Department of Clinical Chemistry, Faculty of Allied Health Sciences, Chulalongkorn University, Bangkok, Thailand; 5 Department of Clinical Chemistry, Faculty of Allied Health Sciences, Chulalongkorn University, Bangkok, Thailand

**Keywords:** Bile duct cancer, Cell death, Metabolomics

## Abstract

Cholangiocarcinoma (CCA) is a bile duct malignancy. Our previous comprehensive analysis showed that ferroptosis-related genes can stratify CCA patients into low-risk and high-risk groups based on survival time. Here, we explored the role of ferroptosis in CCA by analyzing mRNA expression in CCA patients from public databases. We identified acyl-CoA synthetase long chain family member 3 (ACSL3) as a potential ferroptosis suppressor in high-risk CCA patients. Using a panel of CCA cell lines, we confirmed ACSL3 upregulation in CCA cell lines associated with high-risk CCA, correlating this with resistance to the ferroptosis inducer RSL3. Lipidomic analysis revealed increased monounsaturated fatty acid (MUFA)-containing phospholipids in resistant cell lines. ACSL3 silencing sensitized these cells to RSL3. Resistance to ferroptosis was also dependent on exogenous MUFAs and was enhanced by lipid droplet biogenesis inhibition. These findings highlight ACSL3 as a promising target for therapeutic strategies aimed at overcoming ferroptosis resistance in CCA.

## Introduction

Cholangiocarcinoma (CCA) is a type of cancer that originates from the bile ducts. The incidence of CCA has increased globally in recent years, and CCA contributes significantly to cancer-related deaths, due to its refractoriness to chemotherapy^1^. Despite advances in therapeutic options, such as chemotherapy and immunotherapy, the overall prognosis for CCA patients remains unsatisfactory^2,3^. Consequently, there is an urgent need for novel therapeutic approaches to improve outcomes in CCA. While targeted therapies targeting specific driver mutations in CCA, including fibroblast growth factor receptor 2 (FGFR2) fusions and isocitrate dehydrogenase 1 (IDH1) mutations using FGFR and IDH1 inhibitors, have shown promise, these therapies only benefit a small group of CCA patients. Their efficacy is limited, and resistance remains a significant challenge^4–6^. Due to the complexity of oncogene signaling in cancers, including CCA, targeting a single molecule is often insufficient. Additionally, drugs targeting a single molecule may not be effective due to acquired resistance mechanisms. Therefore, combination therapy coupled with personalized medicine has become a promising approach for more effective treatment in modern cancer therapy^6,7^.

Ferroptosis is a regulated form of cell death characterized by iron-dependent accumulation of lipid peroxides^8,9^. Ferroptosis has emerged as a promising target for cancer therapy due to its potential to selectively eliminate cancer cells^10^. Recent work demonstrated that uptake of oxidized cysteine (cystine) *via* the cystine transporter, system x_c_^-^, is a critical dependency of pancreatic ductal adenocarcinoma, and deletion of the system x_c_^-^ subunit *Slc7a11* inhibited tumor growth in mice^11^. Moreover, resistance to ferroptosis has been identified as a potential mechanism contributing to ineffective cancer treatment^12^. Therefore, a better understanding of ferroptosis resistance is essential for the development of effective therapeutic strategies in cancer.

Ferroptosis can be viewed essentially as a form of “lipotoxicity” as it is contingent on the presence of oxidizable lipids, and lipid peroxidation^13^. Pioneering work identified acyl-CoA synthetase long chain family member 4 (ACSL4) as a positive regulator of ferroptosis. ACSL4 plays a key role in activating polyunsaturated fatty acids (PUFAs) by converting long-chain fatty acids into acyl-CoA esters (PUFA-CoA), which serve as substrates for lipid peroxidation^14,15^. Acyl-CoA synthetase long chain family member 3 (ACSL3), in turn, synthesizes monounsaturated fatty acids (MUFAs), which may inhibit the peroxidation of PUFAs, thereby providing protection against ferroptosis^16^. ACSL3 was recently found to play a role in the sensitivity to ferroptosis in clear cell renal cell carcinoma^17^.

We previously classified CCA patients on the basis of a ferroptosis-related gene signature, stratifying them into low-risk and high-risk groups according to survival outcomes^18^. Extending this classification to CCA cell lines, we found that the cell lines corresponding to the high-risk group of patients exhibited resistance to ferroptosis^18^. Therefore, understanding the underlying mechanisms of ferroptosis resistance in high-risk CCA is crucial for developing targeted therapeutic approaches to overcome treatment resistance and improve patient outcomes. In this study, we conducted a comprehensive investigation of the expression of ferroptosis suppressor genes in CCA patients using publicly available datasets. Our analysis identified *ACSL3* as a potential ferroptosis suppressor in CCA. Moreover, we found a correlation between ACSL3 expression, ferroptosis resistance, and increased levels of MUFA-containing phospholipids (MUFA-PLs) in CCA cell lines, and demonstrated that ACSL3-mediated ferroptosis resistance in CCA cells is dependent on exogenous MUFAs. We also noted that lipid droplets modulated the sensitivity to ferroptosis in CCA cells. These findings shed light on the role of ferroptosis in CCA, and identify ACSL3 as a key ferroptosis suppressor, and suggest that the ACSL3-mediated ferroptosis resistance pathway is a potential therapeutic target in high-risk CCA patients.

## Results

### ACSL3 is an unfavorable prognostic marker and potential ferroptosis suppressor in CCA

**Fig. 1:**
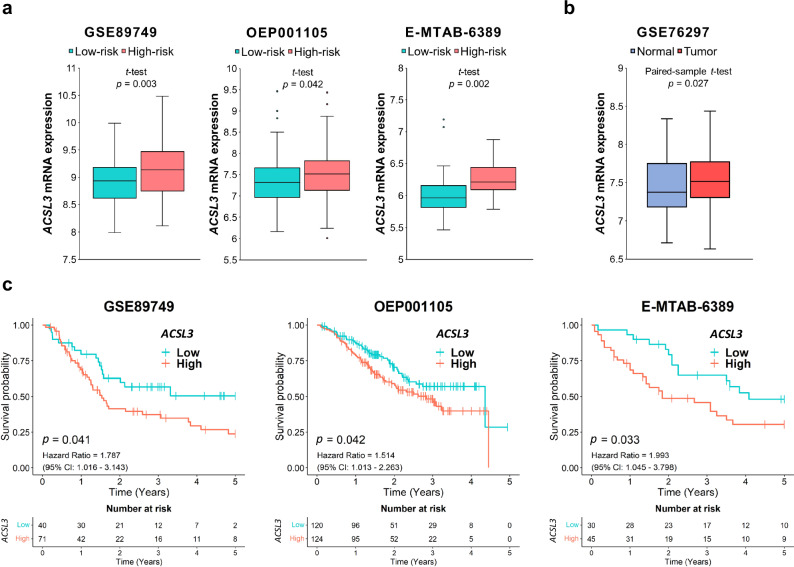
*ACSL3* mRNA expression is significantly upregulated in high-risk CCA patients and serves as an unfavorable prognostic marker. **a** *ACSL3* mRNA expression in CCA tumor tissues of high-risk CCA patients and low-risk CCA patients in GSE89749, OEP001105, and E-MTAB-6389 cohorts. **b** *ACSL3* mRNA expression in CCA tumor tissues and paired-normal bile duct tissues from GSE76297. **c** Kaplan-Meier 5-year survival curves in CCA patients with low and high expression of *ACSL3* in GSE89749, OEP001105, and E-MTAB-6389 cohorts.

To identify a potential ferroptosis suppressor mechanism in CCA, we investigated well-known anti-ferroptotic pathways^19^ across risk groups of CCA patients. To this end, we employed the same patient cohort that was utilized for constructing the ferroptosis-related gene signature^18^. Our findings revealed that among known ferroptosis inhibitory genes, *ACSL3* was significantly upregulated in the high-risk group compared to the low-risk group of CCA patients in the GSE89749 cohort, as well as in two independent cohorts, OEP001105 and E-MTAB-6389 (Fig. 1a). Other ferroptosis inhibitory genes did not display significant differences in expression between high-risk and low-risk CCA patients (Supplementary Fig. 1). Moreover, no differences between the two groups of patients were noted for *ACSL4* (Supplementary Fig. 2). In a previous study, low ACSL3 expression was documented in a small cohort of CCA patients^20^. However, we observed that CCA tissues exhibited a higher level of *ACSL3* compared to adjacent normal tissues (Fig. 1b). Importantly, elevated *ACSL3* expression correlated with shorter survival in CCA patients in the GSE89749, OEP001105, and E-MTAB-6389 cohorts (Fig. 1c). Hence, these findings implicate ACSL3 as a potential ferroptosis suppressor in CCA, as it is upregulated in CCA tissues, and its expression is associated with shorter survival in CCA patients.

### Upregulation of ACSL3 is associated with ferroptosis resistance in human CCA cell lines

**Fig. 2:**
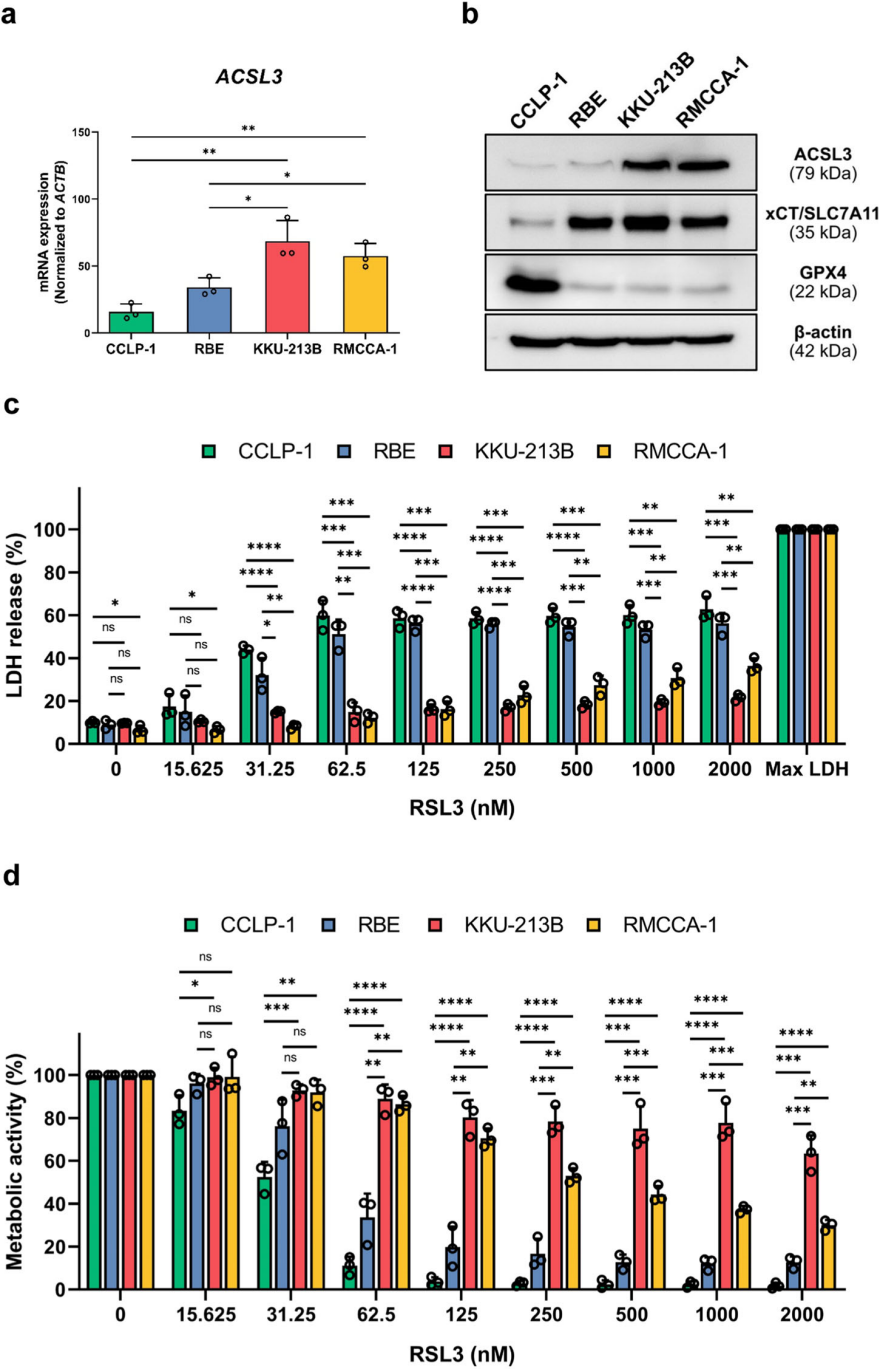
ACSL3 is upregulated in high-risk CCA cell lines and correlates with ferroptosis resistance. **a** *ACSL3* mRNA expression in low-risk CCA cell lines (CCLP-1 and RBE) and high-risk CCA cell lines (KKU-213B and RMCCA-1). **b** ACSL3, xCT/SLC7A11, and GPX4 expression in low-risk CCA cell lines (CCLP-1 and RBE) and high-risk CCA cell lines (KKU-213B and RMCCA-1). **c,** **d** CCA cell lines were exposed for 24 h to RSL3 at the indicated concentrations and cell death/cell viability was determined using the LDH release assay (**c**) and alamarBlue™ (**d**). The data in panels (**c**) and (**d**) are shown as mean values ± S.D. and *p*-values were calculated using two-tailed unpaired Student’s *t*-test. ns, not significant; *p < 0.05; **p < 0.01; ***p < 0.001; ****p < 0.0001.

We previously reported that human CCA cell lines could be classified according to a ferroptosis-related gene signature^18^. Thus, the CCLP-1 and RBE cell lines were classified as “low-risk” whereas the KKU-213B and RMCCA-1 cell lines were classified as “high-risk”. To investigate the association between ACSL3 expression and ferroptosis sensitivity of these cell lines, mRNA and protein levels were examined. We found that both ACSL3 mRNA and protein expression were significantly upregulated in the high-risk cell lines (KKU-213B and RMCCA-1) when compared to the low-risk cell lines (CCLP-1 and RBE) (Fig. 2a, b). However, expression of the well-known ferroptosis inhibitory factors, glutathione peroxidase 4 (GPX4) and xCT/SLC7A11, did not distinguish between high-risk and low-risk CCA cell lines (Fig. 2b). Next, ferroptosis sensitivity was investigated by using the small molecule ferroptosis inducer, RSL3. Cell death/cell viability was monitored by using the LDH release assay and alamarBlue™ assay for metabolic activity. CCA cell lines with high ACSL3 expression (KKU-213B and RMCCA-1) showed greater resistance to RSL3 compared to the CCA cell lines with low ACSL3 expression (CCLP-1 and RBE) (Fig. 2c, d). To confirm ferroptotic cell death, various cell death inhibitors including the antioxidant, ferrostatin-1 (Fer-1), and the iron-chelating agent, deferoxamine (DFO), as well as zVAD-fmk (a pan-caspase inhibitor), and necrosulfonamide (NSA) (an inhibitor of MLKL-RIP1-RIP3 necrosome complex formation), were applied. Only the ferroptosis inhibitors (Fer-1 and DFO) could prevent RSL3-triggered cell death in CCA cell lines (Supplementary Fig. 3a–d). Furthermore, lipid peroxidation was evaluated by flow cytometry by using the fluorescent BODIPY™ 581/591 C11 sensor. RSL3 increased the levels of lipid peroxides, and this was suppressed by Fer-1 and DFO (Supplementary Fig. 3e–h). We also examined the morphology of CCLP-1 cells exposed to RSL3. RSL3-treated cells exhibited smaller mitochondria and an increased density of mitochondrial cristae as well as dilation of the endoplasmic reticulum (ER) when compared to control cells (Supplementary Fig. 4a, b). These findings are largely concordant with previous studies on erastin-treated HT-1080 cells^21^. Mitochondrial remodeling has been implicated in ferroptosis^22^. It is also worth noting that the density of mitochondrial cristae is dynamically altered in skeletal muscle cells to increase the rate of respiration^23^. In sum, the present results suggest that ACSL3 plays a key role as a ferroptosis inhibitory factor/suppressor in CCA cell lines.

### Ferroptosis-resistant cell lines display elevated levels of MUFA-containing phospholipids

**Fig. 3:**
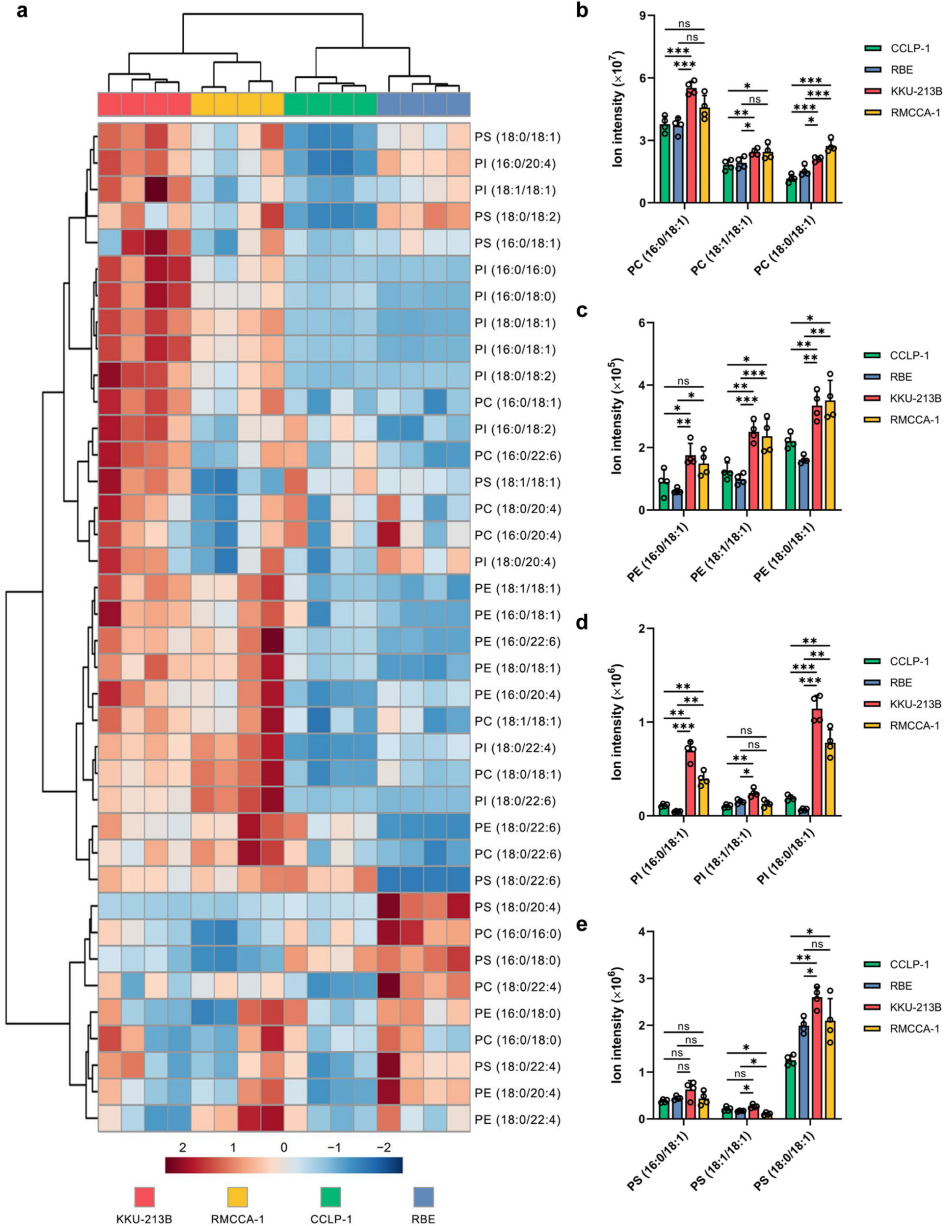
MUFA-containing phospholipid species are elevated in ferroptosis-resistant CCA cell lines. **a** Heatmap showing clustering of the phospholipid species detected by mass spectrometry in CCA cell lines (each cell line represented by four biological replicates). The color scale displays normalized scaling (mean-centered and divided by the standard deviation of each variable). **b**–**e** The levels of the OA-containing phospholipids were determined in the CCA cell lines corresponding to low-risk (CCLP-1 and RBE) and high-risk (KKU-213B and RMCCA-1) groups. The data in (**b**–**e**) are shown as mean values ± S.D. and *p*-values were calculated using two-tailed unpaired Student’s *t*-test. ns, not significant; *p < 0.05; **p < 0.01; ***p < 0.001.

ACSL3 plays a role in lipid metabolism by preferentially utilizing fatty acids, particularly MUFAs, for fatty acyl-CoA synthesis, which is essential for phospholipid synthesis^16,24^. We hypothesized that CCA cell lines with elevated ACSL3 expression would exhibit higher levels of MUFA-PLs as compared to low ACSL3-expressing CCA cell lines. To address this hypothesis, we conducted an untargeted lipidomic analysis of the two groups of CCA cell lines. Principal component analysis (PCA) of the lipidome profiles of the untreated cell lines indicated there was no clear distinction between cell lines in the positive ion mode while distinct differences between the CCA cell lines were evident in the negative ion mode insofar as the high-risk and low-risk cell line formed two distinct clusters (Supplementary Fig. 5a, b). Hierarchical clustering of the indicated phospholipids by their normalized peak area revealed two main clusters of CCA cell lines, corresponding to the high-risk and low-risk CCA cell lines (Fig. 3a). Moreover, we found significant differences between the high-risk and low-risk cell lines insofar as the ferroptosis-resistant (high-risk) CCA cell lines exhibited elevated levels of oleic acid (OA)-containing phospholipids (16:0/18:1, 18:1/18:1, and 18:0/18:1) (Fig. 3b–e). These findings confirm distinct phospholipid profiles between the two groups of CCA cell lines.

### Silencing of ACSL3 expression sensitizes the CCA cell lines to RSL3-induced ferroptosis

**Fig. 4:**
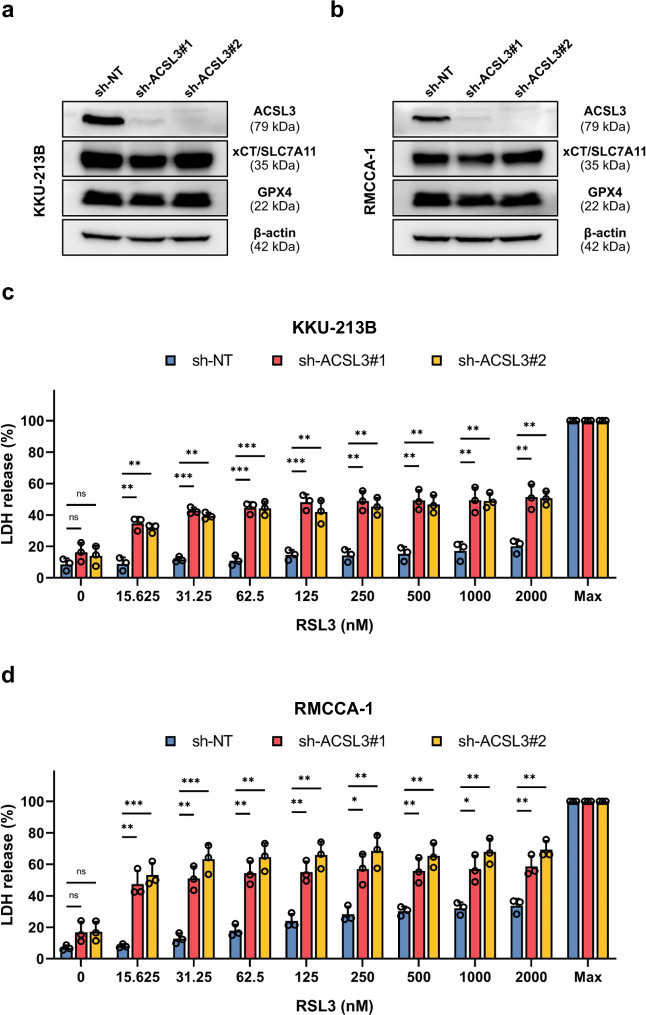
ACSL3 silencing enhances RSL3 sensitivity in resistant CCA cell lines. **a,** **b** Expression of ACSL3, xCT/SLC7A11, and GPX4 was assessed in ACSL3-silenced KKU-213B cells (**a**) and RMCCA-1 cells (**b**). **c,** **d** ACSL3-expressing and ACSL3-silenced KKU-213B cells (**c**) and RMCCA-1 cells (**d**) were exposed for 24 h to RSL3 at the indicated concentrations, and cell death was determined using the LDH release assay. The data in panels (**c**) and (**d**) are shown as mean values ± S.D. and *p*-values were calculated using two-tailed unpaired Student’s *t*-test. ns, not significant; *p < 0.05; **p < 0.01; ***p < 0.001.

**Fig. 5:**
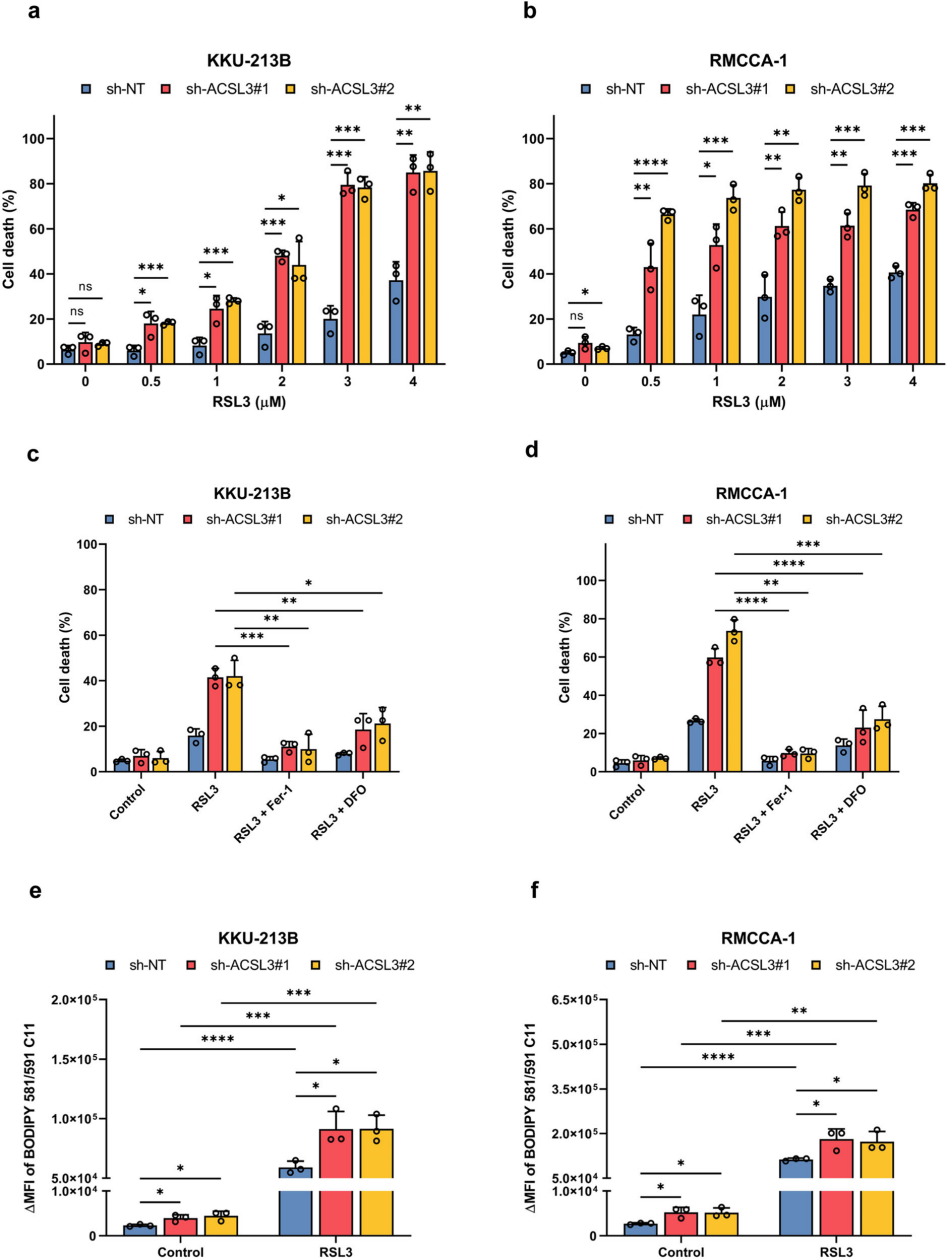
ACSL3 silencing sensitizes resistant CCA cell lines to ferroptosis. **a,** **b** ACSL3-expressing and ACSL3-silenced KKU-213B cells (**a**) and RMCCA-1 cells (**b**) were exposed for 48 h to RSL3 at the indicated concentrations, and cell death was determined based on Annexin V/PI staining. **c,** **d** ACSL3 expressing and ACSL3 silenced KKU-213B cells (**c**) and RMCCA-1 cells (**d**) pretreated with or without Fer-1 (5 µM) or DFO (10 µM) were exposed for 48 h to RSL3 (2 µM for KKU-213B, and 1 µM for RMCCA-1) and cell death was determined based on Annexin V/PI staining. **e,** **f** ACSL3-expressing and ACSL3-silenced KKU-213B cells (**e**) and RMCCA-1 cells (**f**) exposed to medium alone or to RSL3 as detailed above, and lipid peroxidation was determined using the fluorescent BODIPY™ 581/591 C11 sensor. The data are shown as mean values ± S.D. and *p*-values were calculated using two-tailed unpaired Student’s *t*-test. ns, not significant; *p < 0.05; **p < 0.01; ***p < 0.001; ****p < 0.0001.

To investigate the role of ACSL3 in CCA, we selected the ferroptosis-resistant CCA cell lines with high ACSL3 expression (KKU-213B and RMCCA-1) for further studies. First, we stably expressed shRNAs against *ACSL3* in these cells to downregulate the expression of ACSL3 (Fig. 4a, b). The expression of GPX4 and xCT/SLC7A11 remained unaffected, as shown by Western blot. No significant differences in cell proliferation rates were observed between ACSL3-expressing and ACSL3-silenced cells within the first 48 h (Supplementary Fig. 6). Hence, all subsequent experiments using these cells were conducted within a 48 h timeframe. We demonstrated that CCA cell lines with silencing of ACSL3 expression exhibited markedly increased susceptibility to RSL3 compared to ACSL3-expressing cells, as shown by using the LDH release assay (Fig. 4c, d). These findings were corroborated by using the annexin V/propidium iodide (PI) staining assay (Fig. 5a, b). We confirmed that the cell death was inhibitable by Fer-1 and DFO (Fig. 5c, d). Furthermore, the baseline levels of lipid peroxides were significantly elevated in ACSL3 knockdown cells when compared to ACSL3-expressing cell lines, as were the levels of lipid peroxides following RSL3 treatment (Fig. 5e, f). These findings demonstrated that ACSL3 suppresses ferroptosis in high-risk CCA cell lines.

### Exogenous oleic acid is essential for ACSL3-mediated ferroptosis resistance in CCA cells

**Fig. 6:**
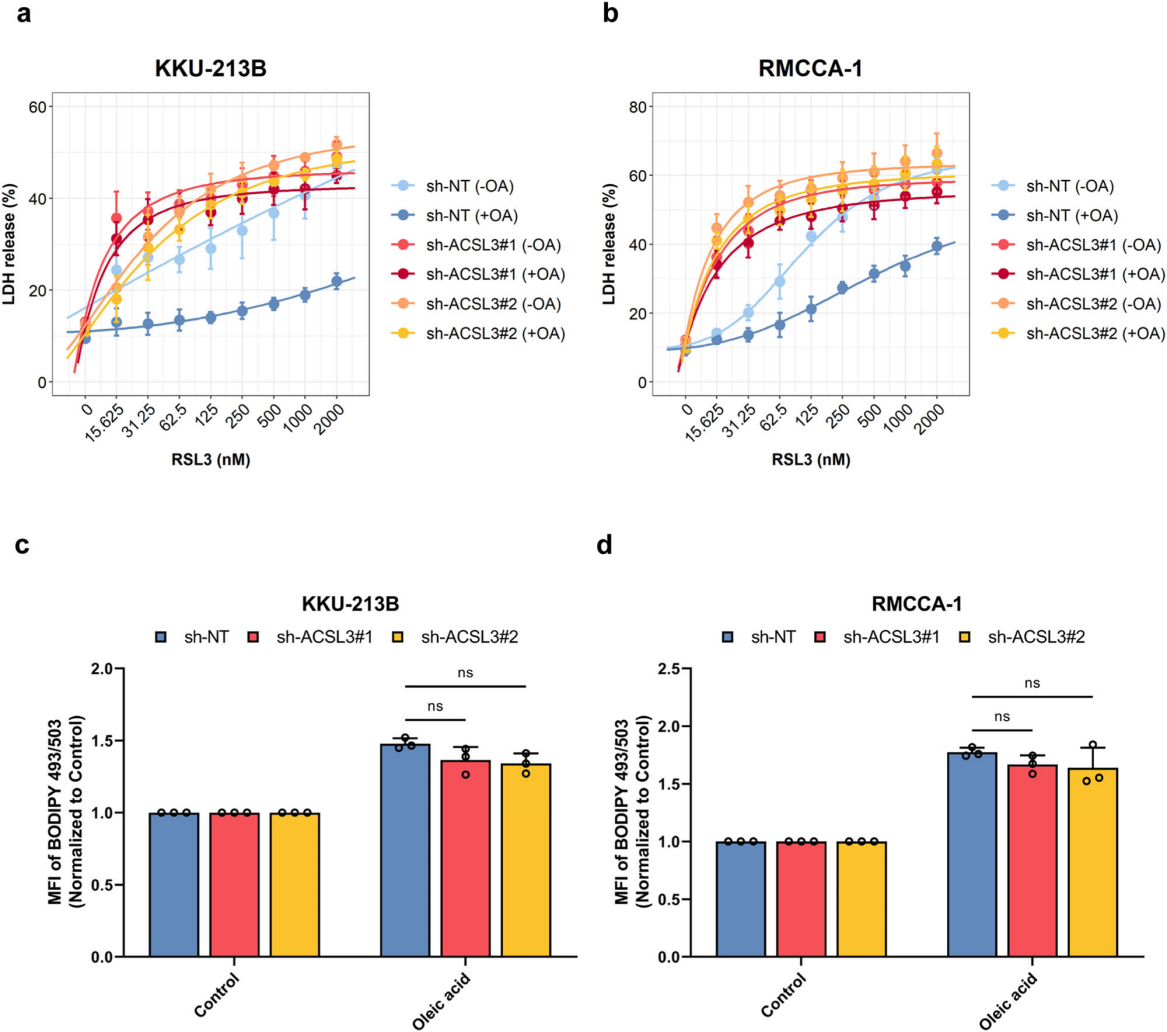
Exogenous oleic acid is essential for ACSL3 to protect against ferroptosis. **a,** **b** ACSL3-expressing and ACSL3-silenced KKU-213B cells (**a**) and RMCCA-1 cells (**b**) were cultured in OA-containing medium or OA-depleted medium and subsequently exposed to RSL3 at the indicated concentrations. Cell death was assessed after 24 h using the LDH release assay. **c**, **d** Quantification of lipid droplets in ACSL3-expressing and ACSL3-silenced KKU-213B cells (**c**) and RMCCA-1 cells (**d**) after exposure for 24 h to OA (100 µM) by flow cytometric detection of BODIPY™ 493/503 stained cells. MFI values are reported. The data in panels (**c**) and (**d**) are shown as mean values ± S.D. and *p*-values were calculated using two-tailed unpaired Student’s *t*-test. ns, not significant.

Exogenous MUFAs serve as substrates for ACSL3-mediated fatty acyl-CoA synthesis, and the major MUFA in the diet is oleic acid (OA) (18:1)^16^. To explore the role of exogenous OA in ACSL3-mediated ferroptosis protection, we cultured ferroptosis-resistant CCA cell lines in either OA-depleted or OA-containing media. We found that the depletion of OA sensitized ACSL3-expressing CCA cell lines to RSL3 compared to cells grown in OA-containing media, while no such differences were observed in the ACSL3-knockdown cell lines (Fig. 6a, b). Given that ACSL3 has been implicated in lipid droplet formation in certain cell types^25,26^, we further investigated the effect of ACSL3 knockdown on lipid droplet formation. However, although the levels of lipid droplets in ACSL3 knockdown cells were slightly decreased, the difference was not significant compared to ACSL3-expressing cells with or without the addition of OA (Fig. 6c, d). Taken together, these findings demonstrated the importance of exogenous OA in ACSL3-mediated ferroptosis resistance in CCA cell lines. However, lipid droplet formation was found not to be affected by the knockdown of ACSL3 expression in CCA cell lines.

### ACSL3 localizes to lipid droplets in CCA cells in response to replenishment of oleic acid

**Fig. 7:**
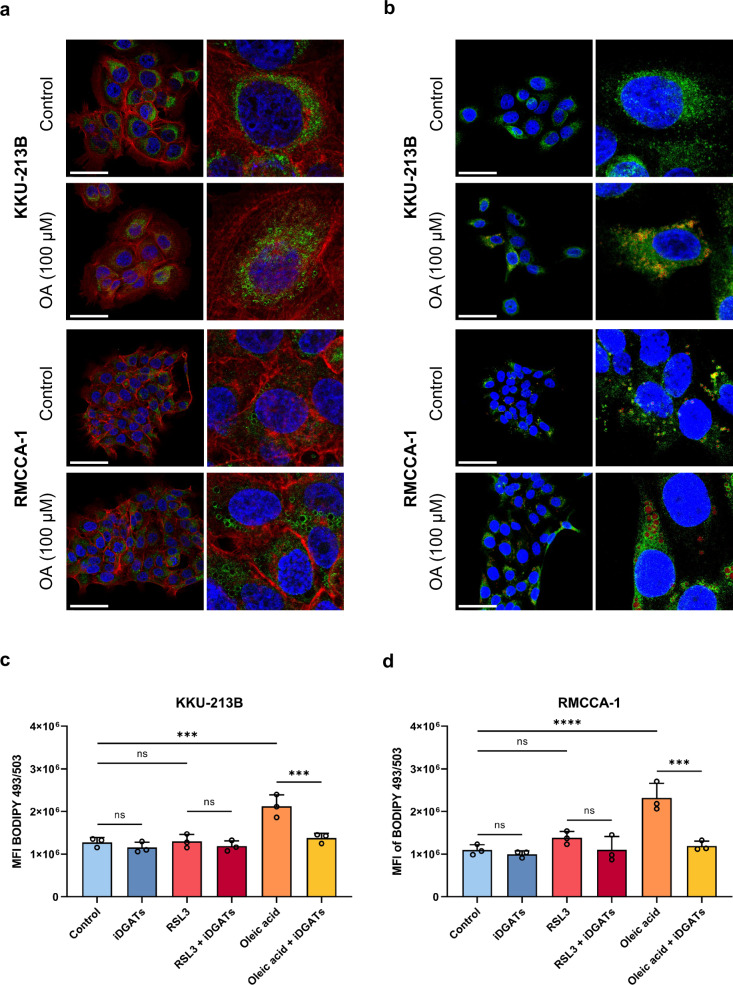
Localization of ACSL3 in ferroptosis-resistant CCA cell lines. **a** Confocal imaging of ACSL3 localization in KKU-213B and RMCCA-1 cells after exposure to vehicle control or OA (100 µM) for 24 h. Cells were stained with antibodies specific for ACSL3 (green) and counterstained with Hoechst 33342 (blue) (cell nuclei) and phalloidin-iFluor 647 (red) (actin filaments). Scale bars: 50 μm. **b** Co-localization studies of ACSL3 and lipid droplets were performed in KKU-213B and RMCCA-1 cells after exposure to vehicle control or OA (100 µM) for 24 h. Cells were stained with antibodies against ACSL3 (green) and cell nuclei were counterstained with Hoechst 33342 (blue), while lipid droplets were stained using Nile Red (red). Scale bars: 50 μm. **c,** **d** Lipid droplet levels in CCA cell lines KKU-213B (**c**) and RMCCA-1 (**d**) exposed to vehicle control, RSL3 or OA in the presence or absence of DGAT1/2 inhibitors for 24 h were quantified based on flow cytometric analysis of BODIPY™ 493/503 staining. The data in panels (**c**) and (**d**) are shown as mean values ± S.D. and *p*-values were calculated using two-tailed unpaired Student’s *t*-test. ns, not significant; ***p < 0.001; ****p < 0.0001. OA, oleic acid.

ACSL3 has been shown to be localized to the ER membrane and lipid droplets, depending on the cell type and on the supply of fatty acids^27^. However, there is a lack of information regarding the localization of ACSL3 in CCA cells, which is crucial for understanding its functional role in ferroptosis resistance. To address this, we investigated the localization of ACSL3 in the ferroptosis-resistant CCA cell lines. First, at baseline conditions, ACSL3 was observed to be perinuclear in KKU-213B and predominantly associated with small vesicles in the cytoplasm (likely lipid droplets) in RMCCA-1 (Fig. 7a and Supplementary Fig 7a). Furthermore, upon OA treatment, ACSL3 was found to localize predominantly to cytoplasmic vesicles/lipid droplets in both cell lines, indicating a dynamic redistribution in response to changes in lipid composition (Fig. 7a and Supplementary Fig 7a). To corroborate the suggestion or assumption that the cytoplasmic vesicles were lipid droplets, we stained the cells using Nile Red (9-diethylamino-5H-benzo[alpha]phenoxazine-5-one) (Fig. 7b and Supplementary Fig 7b).

**Fig. 8:**
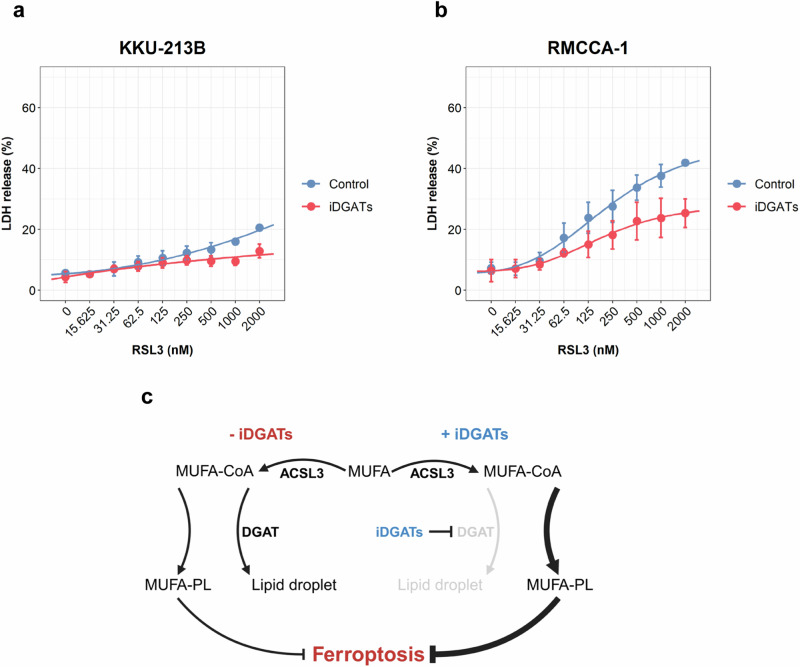
Inhibition of DGAT1 and DGAT2 further aggravates ferroptosis resistance in CCA cell lines. **a,** **b** KKU-213B cells (**a**) and RMCCA-1 cells (**b**) were pretreated with vehicle control or DGAT1 and DGAT2 inhibitors for 24 h, then exposed to RSL3 at the indicated concentrations for 24 h in the presence or absence of DGAT1 and DGAT2 inhibitors. Cell death was assessed using the LDH release assay. **c** Schematic illustration of the effect of DGAT inhibition on ferroptosis sensitivity in the current model. In the absence of iDGATs, exogenous MUFA can be converted into MUFA-CoA by ACSL3. The resulting MUFA-CoA can then be utilized for triglyceride synthesis, leading to lipid droplet formation *via* DGAT or for phospholipid synthesis. However, in the presence of iDGATs, MUFA-CoA cannot be used for triglyceride synthesis. As a result, the available MUFA-CoA is redirected towards MUFA-phospholipid (MUFA-PL) synthesis, which enhances ferroptosis resistance in ACSL3-high-expressing CCA cell lines. iDGATs, DGAT1&2 inhibitors; LD, lipid droplet; MUFA, monounsaturated fatty acid; MUFA-CoA, monounsaturated fatty acyl-coenzyme A; MUFA-PL, monounsaturated fatty acid-containing phospholipid.

Previous work suggested that ACSL3 regulates lipid droplet biogenesis and ferroptosis sensitivity in clear cell renal cell carcinoma^17^. We therefore addressed whether lipid droplet formation was required for ACSL3-mediated ferroptosis resistance in the high-risk CCA cell lines. To inhibit the formation of lipid droplets, the synthesis of triacylglycerols (TAGs) was blocked by simultaneously inhibiting diacylglycerol O-acyltransferase (DGAT) 1 and 2^28^. To monitor lipid droplet formation, cells were stained using the fluorescent neutral lipid dye, BODIPY™ 493/503. We found that OA promoted the formation of lipid droplets, and this was prevented by DGAT1/2 inhibition, whereas RSL3 did not influence lipid droplet formation (Fig. 7c, d). Moreover, the inhibition of lipid droplet formation further increased the resistance to RSL3 in both ferroptosis-resistant cell lines (KKU-213B and RMCCA-1) (Fig. 8a, b), thus suggesting that the reduction of lipid droplet biogenesis might increase ferroptosis resistance (Fig. 8c). In sum, these findings suggest that exogenous OA is essential for ACSL3-mediated protection against ferroptosis, and we could also demonstrate the dynamic redistribution of ACSL3 to lipid droplets in ferroptosis-resistant CCA cells replenished with OA. However, ferroptosis induction using RSL3 did not seem to affect the formation of lipid droplets in CCA cells.

## Discussion

In our previous study, we classified CCA patients into low-risk and high-risk groups based on ferroptosis-related genes, revealing poorer survival outcomes in the high-risk group^18^. Extending this classification to CCA cell lines, we observed that cell lines corresponding to the high-risk patient group exhibited resistance to ferroptosis^18^. However, the specific regulatory mechanisms of ferroptosis linking survival outcomes and resistance in CCA remain unknown. In this study, we identified ACSL3 as a putative ferroptosis suppressor in CCA, with elevated expression observed in high-risk group CCA patients correlating with shorter survival time. Furthermore, we could show that high-risk (ferroptosis-resistant) CCA cell lines displayed higher ACSL3 expression when compared to low-risk CCA cell lines, and silencing of ACSL3 expression re-sensitized the high-risk CCA cell lines to ferroptosis, thus confirming the role of ACSL3 in ferroptosis resistance. These results show that ACSL3 is an unfavorable prognostic marker in CCA, and identify ACSL3 as a key regulator of ferroptosis sensitivity in CCA derived cell lines.

Upregulation of the transferrin receptor and accumulation of iron were previously observed in CCA tissues, and this correlated with poor prognosis in CCA patients^29^. Furthermore, iron metabolism related genes were found to be dysregulated in CCA stem cell-like cells^30^. These findings support the idea that targeting ferroptosis could represent a viable therapeutic strategy for CCA. The SLC7A11/GPX4 axis is one of the main antioxidant defense systems in ferroptosis^31^. Moreover, it has been shown that p53 inhibits cystine uptake and sensitizes cells to ferroptosis by repressing the expression of *SLC7A11*^32^. Several studies addressed the potential role of the p53/SLC7A11/GPX4 pathway in CCA^33–36^. However, we found that the expression of SLC7A11 and GPX4 did not correlate with ferroptosis sensitivity in CCA cell lines. Instead, we have shown that ACSL3 expression is correlated with resistance to ferroptosis.

ACSL3 is involved in lipid metabolism by using MUFAs, for the synthesis of fatty acyl-CoA, which is crucial for phospholipid synthesis^16^. The present lipidomic analysis revealed greater incorporation of OA into phosphatidylinositol (PI) and phosphatidylethanolamine (PE), as well as in phosphatidylcholine (PC) and to a lesser extent in phosphatidylserine (PS), in high-risk CCA cell lines when compared to their low-risk counterparts. This difference in OA incorporation between high-risk and low-risk cell lines was especially notable for PI, which is a relatively less abundant phospholipid in comparison to PC, PE, and PS. It is interesting to consider that while AA-PE and AA-PC are well-established substrates of lipid peroxidation in ferroptosis^9^, the PI subclass has received less attention. However, a recent study demonstrated that heightened levels of AA-PI may promote ferroptosis susceptibility in ovarian and renal carcinoma cell lines^37^. Notwithstanding, our results showed that ferroptosis-resistant and ferroptosis-sensitive cell lines could be segregated on the basis of the incorporation of OA into cellular phospholipids.

Recent work has shown that OA also blocks iron-overload-induced lipid peroxidation^38^. These findings are relevant for cancer therapy. Indeed, a recent study demonstrated that OA enabled melanoma cells to evade ferroptosis in an ACSL3-dependent manner^39^. The authors noted that ACSL3 was expressed by most human and mouse melanomas, including all of the efficient metastasizers whereas some of the inefficient metastasizers expressed little ACSL3. The present study demonstrated an association between elevated ACSL3 expression, heightened levels of MUFA-PLs, and ferroptosis resistance in CCA cells. Therefore, targeting ACSL3 is a promising therapeutic strategy in CCA. More generally, metabolic and genetic profiling has proven a fruitful approach in difficult-to-treat cancers^40^.

ACSL3-dependent conversion of exogenous MUFAs to fatty acyl-CoA for further synthesis of phospholipids was previously shown to promote ferroptosis resistance in HT-1080 cells^16^. ACSL3 is located at both the ER and on lipid droplets, and it has been suggested that lipid droplets together with the surrounding ER membranes form a functional unit^27,41^. Do lipid droplets play a role in ferroptosis? It is conceivable that lipid droplet formation could protect cells from lipotoxicity by sequestering PUFAs, which serve as substrates for lipid peroxidation in ferroptosis. On the other hand, lipid droplets could also serve as a source of oxidizable phospholipids, being released through autophagic degradation or “lipophagy”^42^. Interestingly, lipid droplet formation was ruled out in the ACSL3-dependent protection against ferroptosis in the aforementioned study^16^. Moreover, using the same cell line (HT-1080), other investigators have shown that lipid droplets do not play a role in the prevention or promotion of ferroptosis, even though PUFA incorporation in lipid droplets was observed^28^. On the other hand, using a renal cell carcinoma cell line, cell cycle arrest was found to induce DGAT-dependent lipid droplet formation and resistance to ferroptosis^43^. Moreover, the authors could show that DGAT inhibitors synergized with ferroptosis inducers to overcome therapy resistance in a human colorectal carcinoma xenograft model. In the present study, we found that supplementation with exogenous OA promoted the formation of lipid droplets in ferroptosis-resistant CCA cell lines. We also found that ACSL3 appeared to be predominantly redistributed to lipid droplets in CCA cells upon supplementation with OA (by using confocal microscopy). However, DGAT1/2 inhibitors aggravated ferroptosis resistance in the high-risk CCA cell lines, suggesting a role for lipid droplets as potential drivers of ferroptosis in CCA. This finding implies that inhibition of DGAT1/2 might reduce TAG production from acyl-CoA, particularly MUFA-CoA in cells with high ACSL3 expression, thereby increasing the availability of acyl-CoA for MUFA-PL synthesis and promoting ferroptosis resistance. In contrast, previous studies demonstrated that DGAT inhibitors promoted ferroptosis of cancer cells adapted to acidic pH by preventing the protective shunting of PUFAs into lipid droplets^44^. Therefore, it is possible that lipid droplets play different roles in different cellular contexts.

**Fig. 9:**
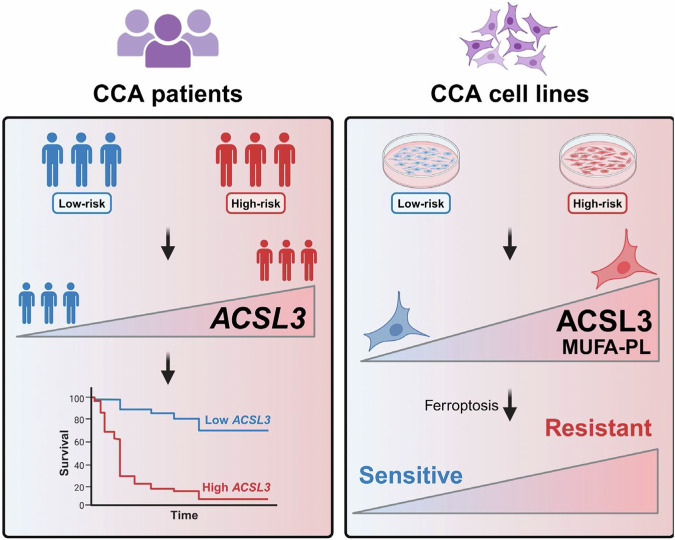
Schematic summary of the role of ACSL3 in CCA patients and cell lines. Elevated expression of *ACSL3* is observed in high-risk group CCA patients, correlating with shorter survival times (left). High-risk CCA cell lines with elevated ACSL3 levels exhibit increased levels of MUFA-PL, which are associated with increased resistance to ferroptosis (right). MUFA-PL: monounsaturated fatty acid-containing phospholipid.

In conclusion, we identified ACSL3 as an unfavorable prognostic marker in CCA patients, demonstrating a significant association between high ACSL3 expression, increased levels of MUFA-PLs, and ferroptosis resistance in CCA cell lines (Fig. 9). By elucidating the role of ACSL3 in ferroptosis resistance, we support the view that ACSL3 is a “gatekeeper” of ferroptosis^45^. These findings provide precise insights into the mechanisms underlying ferroptosis resistance in CCA, highlighting the ACSL3-mediated pathway as a promising target for precision therapeutic strategies aimed at improving survival in high-risk CCA patients with elevated ACSL3 expression.

## Methods

### Data analysis

Four public transcriptomic datasets and clinical information were retrieved from three platforms. The E-MTAB-6389 dataset previously reported by Job et al.^46^ was downloaded from the European Bioinformatics Institute (EMBL-EBI) database (https://www.ebi.ac.uk/). The OEP001105 dataset previously reported by Dong et al.^47^ was downloaded from the National Omics Data Encyclopedia (NODE) database (https://www.biosino.org/node/). The GSE76297 and GSE89749 datasets previously reported by Chaisaingmongkol et al.^48^ and Jusakul et al.^49^ were downloaded from the Gene Expression Omnibus (GEO) database (https://www.ncbi.nlm.nih.gov/geo/). Clinical information for the GSE89749 dataset was obtained from a previous study^49^. Expression of *ACSL3* mRNA in tumor tissues and paired non-tumor tissues was investigated in GSE76297 by using a paired-sample *t*-test. Kaplan-Meier and log-rank test were used to analyze the correlation of *ACSL3* mRNA expression with patient survival in E-MTAB-6389, OEP001105 and GSE89749 datasets using the “survminer” package.

### Cell culture

The CCLP-1, KKU-213B, and RBE cell lines were obtained from the Japanese Collection of Research Bioresources (JCRB) Cell Bank (Osaka, Japan). The RMCCA-1 cell line was developed from a Thai patient with CCA^50^. The HEK293T cell line was obtained from American Type Culture Collection (ATCC). All the CCA cell lines were maintained in RPMI-1640 medium (HyClone Laboratories, or Invitrogen), while HEK293T was maintained in Dulbecco’s modified Eagle medium (DMEM) (Invitrogen), supplemented with 10% fetal bovine serum (FBS) (Sigma-Aldrich) and 1% penicillin-streptomycin (HyClone Laboratories, or Invitrogen). The cell lines were all cultured in a humidified incubator at 37 °C with 5% CO_2_. CCA cell lines and the HEK293T cell line were routinely tested for mycoplasma contamination.

### Gene silencing

The RNAi consortium (TRC) lentiviral human *ACSL3* shRNA plasmids ATTACTGCAATATCTGAGGGC (sh-*ACSL3*#1) and ATCTAAAGTATCACATCCAGG (sh-*ACSL3*#2) were purchased from Dharmacon (Lafayette, CO). To generate lentiviral particles, HEK293T cells were co-transfected with packaging plasmid (pCMV-VSV-G) and envelope plasmid (pCMV-dr8.2-dvpr), along with either shRNA-non-targeting (sh-NT) and sh-*ACSL3*#1 or sh-*ACSL3*#2. After 24 h, supernatants containing viral particles were collected and filtered through a 0.45 μm sterile filter membrane (Merck Millipore). Polybrene (Merck Millipore) was added to the lentiviral preparation at the concentration of 8 μg/mL and then used to infect the cells. After 24 h of infection, the cells were selected with puromycin (Merck Millipore) for 48 h.

### Reverse transcription-quantitative PCR (RT-qPCR)

Total RNA was extracted from the cells using GENEzol™ Reagent (Geneaid Biotech, Taiwan). Subsequently, 1 μg of RNA was reverse-transcribed using Maxime™ RT-PCR PreMix (iNtRON Biotechnology, Seongnam-si, Gyeonggi-do, Republic of Korea). RT-qPCR was performed using iTaq™ Universal SYBR^®^ Green Supermix (Bio-Rad, Hercules, CA, USA) following the manufacturer’s instructions. The specific primers used for RT-qPCR were as follows: *ACSL3* (Forward, 5’ to 3’): AGAGTTTGAACCCGATGGAT, *ACSL3* (Reverse, 5’ to 3’): TCCCAAGTCCCTTTAAGTCC, *ACTB* (β-actin) (Forward, 5’ to 3’): AGAGCTACGAGCTGCCTGAC, and *ACTB* (β-actin) (Reverse, 5’ to 3’): AGCACTGTGTTGGCGTACAG. *ACTB* was employed as an internal control.

### Western blot

For protein detection, cells were washed twice with cold phosphate-buffered saline (PBS) and were lysed in RIPA buffer (Merck Millipore, Darmstadt, Germany) containing a proteinase inhibitor cocktail (Roche, Mannheim, Germany) on ice for 30 min. Total protein concentrations were determined by Bradford assay (Bio-Rad, Hercules, CA). Total proteins (25–50 μg) were separated by 4–12% SDS-PAGE and proteins were transferred onto PVDF membranes. The membranes were blocked in 5% blotting-grade blocker (Bio-Rad) in TBS-T (Tris-buffered saline, 1% Tween 20) buffer at room temperature for 1 h, and the membranes were incubated with the primary antibodies at 4˚C overnight. The primary antibodies used were anti-ACSL3 (sc-166374) from Santa Cruz Biotechnology (Dallas, TX), anti-GPX4 (ab125066) from Abcam (Cambridge, UK), and anti-xCT/SLC7A11 (12691), and anti-β-actin (4970) from Cell Signaling Technology (Danvers, MA). After incubation with primary antibodies, the membranes were washed three times with TBS-T buffer and incubated with horseradish peroxidase-conjugated secondary antibodies from Cell Signaling Technology at room temperature for 1 h. Proteins were visualized by enhanced chemiluminescence according to the manufacturer’s instructions (Bio-Rad) using the Amersham ImageQuant™ 800 imaging system. All raw data from the Western blot experiments shown in the Figures are provided in Supplementary Fig. 8.

### LC–MS analysis

Cells (1.5 ×10^6^) were seeded into 100 mm cell culture dishes. After 24 h, the cells were washed twice with PBS and scraped in 5 mL PBS, followed by centrifugation at 3000 rpm for 10 min. Cell pellets were resuspended in 1.5 mL PBS. Total lipids were extracted in the mixture of 3 mL chloroform and 1.5 mL methanol, followed by vigorous shaking and centrifugation at 1500 g for 3 min. The lower organic layer was collected and evaporated to dryness under a stream of nitrogen and placed at −20 °C for storage until further use. The extracts were reconstituted in 180 µl of chloroform, and 40 µl of each sample was quantitated on an Ultimate DGP-3600SD LC coupled to a Bruker MicrOTOF Q-II MS instrument both in positive and negative ion modes, as previously described^51^. Phospholipids were identified by accurate masses, retention times, and MS/MS spectra, and by comparing them with those of chemical standards, which included PE (16:0/16:0), PS (16:0/16:0), and PC (16:0/16:0). Principal component analysis (PCA) was performed in R using the “prcomp” package. The heatmap and hierarchical clustering of the abundances of phospholipids was generated using MetaboAnalyst 6.0 (www.metaboanalyst.ca).

### Cell death assays

Cell death was determined as described previously^52^. The lactate dehydrogenase (LDH) release assay was conducted using the CytoTox96^®^ Non-Radioactive Cytotoxicity Kit (Promega, Madison, WI). The absorbance values were measured using the Tecan Infinite^®^ F200 plate reader (Männedorf, Switzerland). The percentage of LDH release was normalized by the maximum LDH release. The metabolic activity of the cells was measured by alamarBlue™ Cell Viability Reagent (Thermo Fisher Scientific, Waltham, MA) according to the manufacturer’s instruction. The absorbance values were measured using the Tecan Infinite^®^ F200 plate reader (Männedorf, Switzerland). The percentage of metabolic activity was normalized by vehicle control. To verify the mechanism of cell death, the following inhibitors were used: RSL3 (RAS-selective lethal compound 3), deferoxamine (DFO) from APExBIO Technology LLC (Houston, TX), ferrostatin-1 (Fer-1) from Sigma-Aldrich (St. Louis, MO), the pan-caspase inhibitor, zVAD-fmk and necrosulfonamide (NSA) from Calbiochem (Merck Millipore, Darmstadt, Germany). Additionally, cell death was determined by flow cytometry. Briefly, cells were collected by trypsinization, washed with PBS, and resuspended in Annexin V binding buffer containing FITC-conjugated recombinant Annexin V (ImmunoTools) and propidium iodide (PI) (Invitrogen). The cells were analyzed using the BD Accuri™ C6 Plus flow cytometer (BD Biosciences, Franklin Lakes, NJ), and data were analyzed and plotted using FlowJo™ software.

### TEM imaging

TEM was performed as described^53^ to visualize ultrastructural changes in CCA cells exposed to RSL3. Cells were treated with DMSO (vehicle control) or RSL3 for 8 h. The cells were then harvested by trypsinization and fixed with 2.5% glutaraldehyde in 0.1 M sodium phosphate buffer, pH 7.4 at room temperature for 1 h and further fixed overnight in the refrigerator. After washing with 0.1 M phosphate buffer, the samples were centrifuged and post-fixed in 2% osmium tetroxide in 0.1 M sodium phosphate buffer, pH 7.4 at 4 °C for 2 h. The cells were dehydrated in ethanol and acetone, then embedded in LX-112 resin (Ladd Research, Essex Junction, VT). Ultrathin sections (50–80 nm) prepared using a Leica EM UC6 microtome and contrasted with uranyl acetate followed by lead citrate were examined using a Hitachi HT7700 120 kV TEM (Hitachi High-Tech, Tokyo, Japan). Digital images were captured using a Veleta 2k×2k side-mounted TEM CCD Camera (Olympus Soft Imaging Solutions, Münster, Germany).

### Fatty acid-BSA

Fatty acids (FAs), including oleic acid (OA), linoleic acid (LA), and palmitic acid (PA) (Sigma-Aldrich), were dissolved in ethanol to prepare a stock solution. The FAs were conjugated with FA-free bovine serum albumin (BSA) in PBS (molar FA:BSA ratio 5:1). The vehicle control was prepared by mixing ethanol with FA-free BSA in PBS. The solutions were stored at −20 °C.

### Lipid peroxidation

To further study the mode of cell death, lipid peroxidation was investigated. To this end, 80,000 cells were seeded into 12-well plates one day before the experiment. Then, cells were treated with DMSO (vehicle control) or RSL3 for 6 h. The cells were then stained with 5 μM of BODIPY™ 581/591 C11 (Thermo Fisher Scientific, Waltham, MA) for 30 min. The cells were harvested by trypsinization and resuspended in PBS, and analyzed using the BD Accuri™ C6 Plus flow cytometer (BD Biosciences), and data were analyzed and plotted using FlowJo™ software. The DGAT1 and DGAT2 inhibitors were both from Sigma-Aldrich (St. Louis, MO).

### Lipid droplet analysis

For the quantification of lipid droplets, 40,000 cells were seeded overnight in 24-well plates. Then, cells were exposed as indicated for 24 h. OA (100 µM) was used to elicit lipid droplets. Following treatment, the cells were harvested by trypsinization and stained with 5 μM BODIPY™ 493/503 (Thermo Fisher Scientific) for 15 min. The stained cells were then analyzed using the BD Accuri™ C6 Plus flow cytometer (BD Biosciences), and data were analyzed and plotted using FlowJo™ software.

### ACSL3 localization

KKU-213B and RMCCA-1 cells were seeded overnight at a density of 40,000 cells on poly-L-lysine-coated coverslips in 24-well plates. The following day, cells were exposed to vehicle control or OA (100 µM) for 24 h. Then, the cells were washed three times with PBS, fixed with 4% paraformaldehyde for 15 min, permeabilized with 0.1% Triton-X 100 for 15 min, and blocked with 3% BSA for 1 h. Samples were then incubated with anti-ACSL3 antibody (H00002181-B01P) (Abnova) at a 1:200 dilution for 1 h at room temperature. Subsequently, coverslips were incubated with anti-mouse-IgG conjugated to Alexa Fluor™ 488 (Thermo Fisher Scientific) at a 1:1000 dilution for 1 h at room temperature. Following antibody incubation, the cells were stained for 15 min with Hoechst 33342 (0.5 μg/mL) (ImmunoChemistry Technologies, Davis, CA) to visualize cell nuclei, along with actin-binding Phalloidin-iFluor 647 (Abcam) (1:200) or Nile Red (0.5 μg/mL) (Sigma-Aldrich) to visualize lipid droplets. Finally, samples were mounted with Prolong™ Diamond Antifade Mountant (Thermo Fisher Scientific) and imaged using a Leica Stellaris 5 X confocal microscope (Leica).

### Statistical analysis

Data were analyzed and visualized using R statistical software (version 4.1.0), SPSS (Version 22.0, IMM Corp, Armonk, NY), or GraphPad Prism 8.0. Two-tailed unpaired Student’s *t*-test was used to assess differences between groups. All results with *p*-values < 0.05 were considered to be statistically significant. For the clinical samples, the log-rank test was used to assess differences in survival, and Kaplan-Meier analyses were performed to compute patient survival.

## Supplementary information

Supplementary material

## Data Availability

The datasets analyzed in this study were retrieved from the following databases: Gene Expression Omnibus (GEO) (https://www.ncbi.nlm.nih.gov/geo/), European Bioinformatics Institute (EMBL-EBI) (https://www.ebi.ac.uk/), and National Omics Data Encyclopedia (NODE) (https://www.biosino.org/node/). The accession numbers are provided in the article. All other data supporting this study are included within the article and/or supporting materials.
