# Supplementary material for: ACSL3 is an unfavorable prognostic marker in cholangiocarcinoma patients and confers ferroptosis resistance in cholangiocarcinoma cells
Source: NPJ Precis Oncol. 2024 Dec 20;8:284. doi: 10.1038/s41698-024-00783-8 (PMC11662031; doi:10.1038/s41698-024-00783-8)
Supplement: Supplementary file 1 — Supplementary material [file 41698_2024_783_MOESM1_ESM.pdf]

Supplementary Information

# ACSL3 is an unfavorable prognostic marker in cholangiocarcinoma patients and confers ferroptosis resistance in cholangiocarcinoma cells

Apiwit Sae-Fung[1,2], Nawaporn Vinayavekhin[3], Bengt Fadeel[2], and Siriporn Jitkaew[4,5,*]

[1]Graduate Program in Clinical Biochemistry and Molecular Medicine, Department of Clinical Chemistry, Faculty of Allied Health Sciences, Chulalongkorn University, Bangkok, Thailand.

[2]Division of Molecular Toxicology, Institute of Environmental Medicine, Karolinska Institutet, Stockholm, Sweden.

[3]Center of Excellence in Natural Products Chemistry, Department of Chemistry, Faculty of Science, Chulalongkorn University, Bangkok

[4]Center of Excellence for Cancer and Inflammation, Department of Clinical Chemistry, Faculty of Allied Health Sciences, Chulalongkorn University, Bangkok, Thailand.

[5]Department of Clinical Chemistry, Faculty of Allied Health Sciences, Chulalongkorn University, Bangkok, Thailand.

[*]Corresponding author. E-mail: siriporn.ji@chula.ac.th

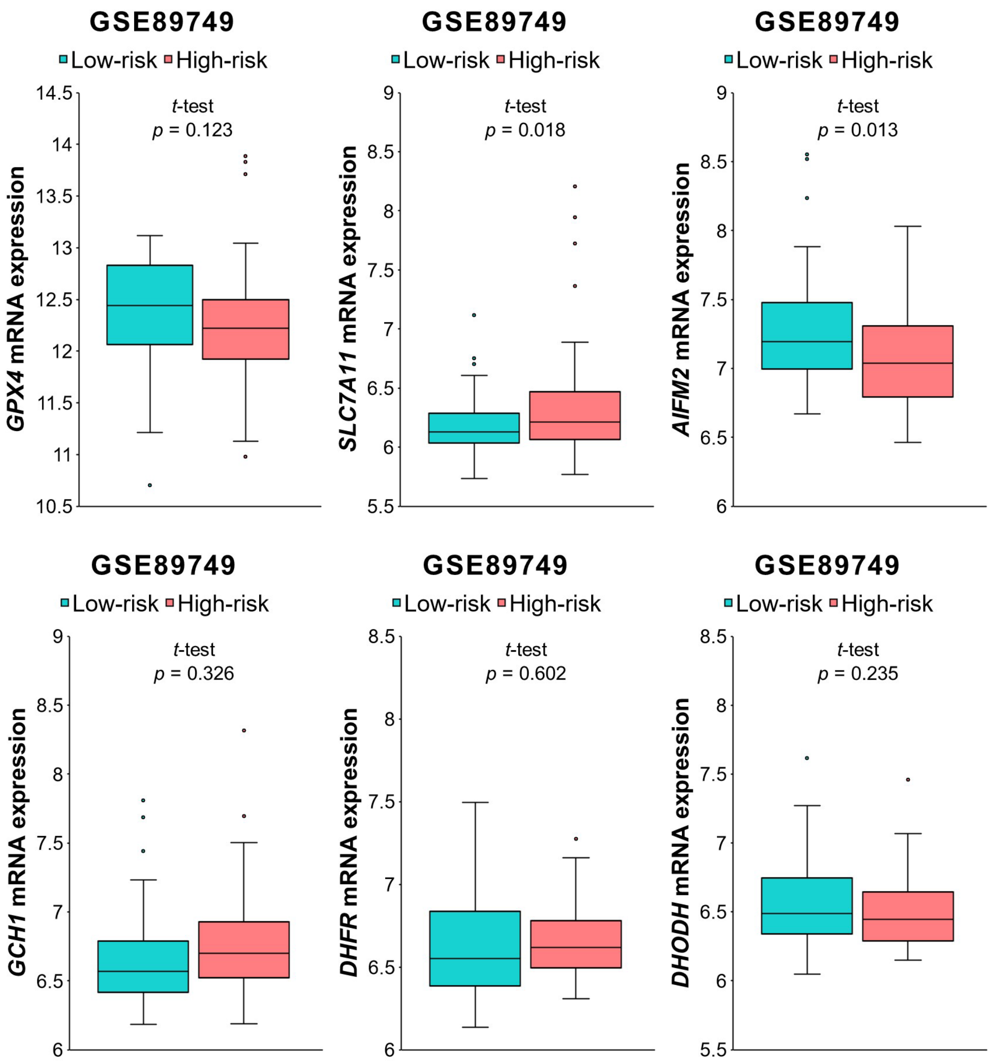


Supplementary Fig. 1 Gene expression in CCA patients. Box plots of ferroptosis inhibitory gene mRNA expression in CCA tumor tissues of high-risk CCA patients and low-risk CCA patients from the GSE89749 cohort (n = 111).

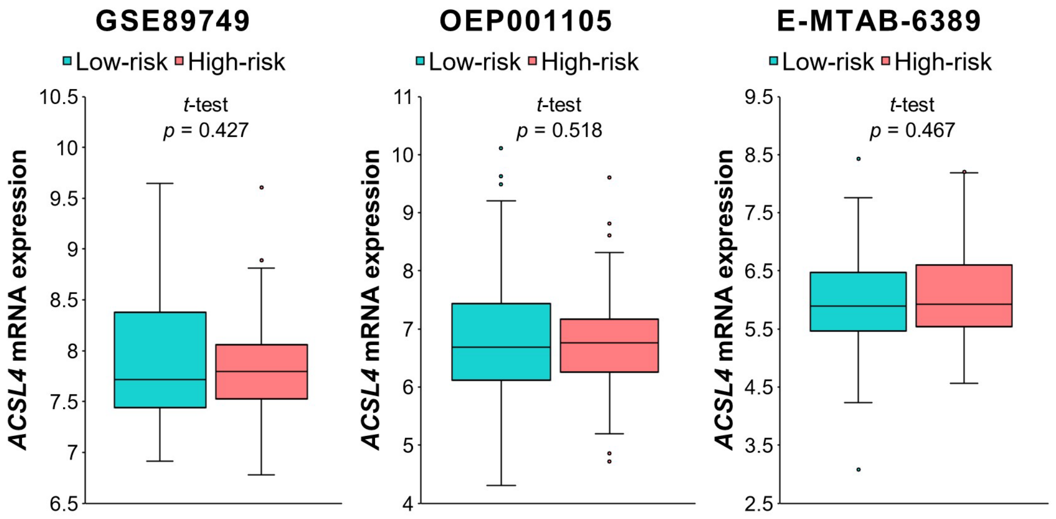


Supplementary Fig. 2 *ACSL4* mRNA expression in CCA tumor tissues of high-risk CCA patients and low-risk CCA patients from the GSE89749 (n = 111), OEP001105 (n = 244), and E-MTAB-6389 (n = 75) cohorts.

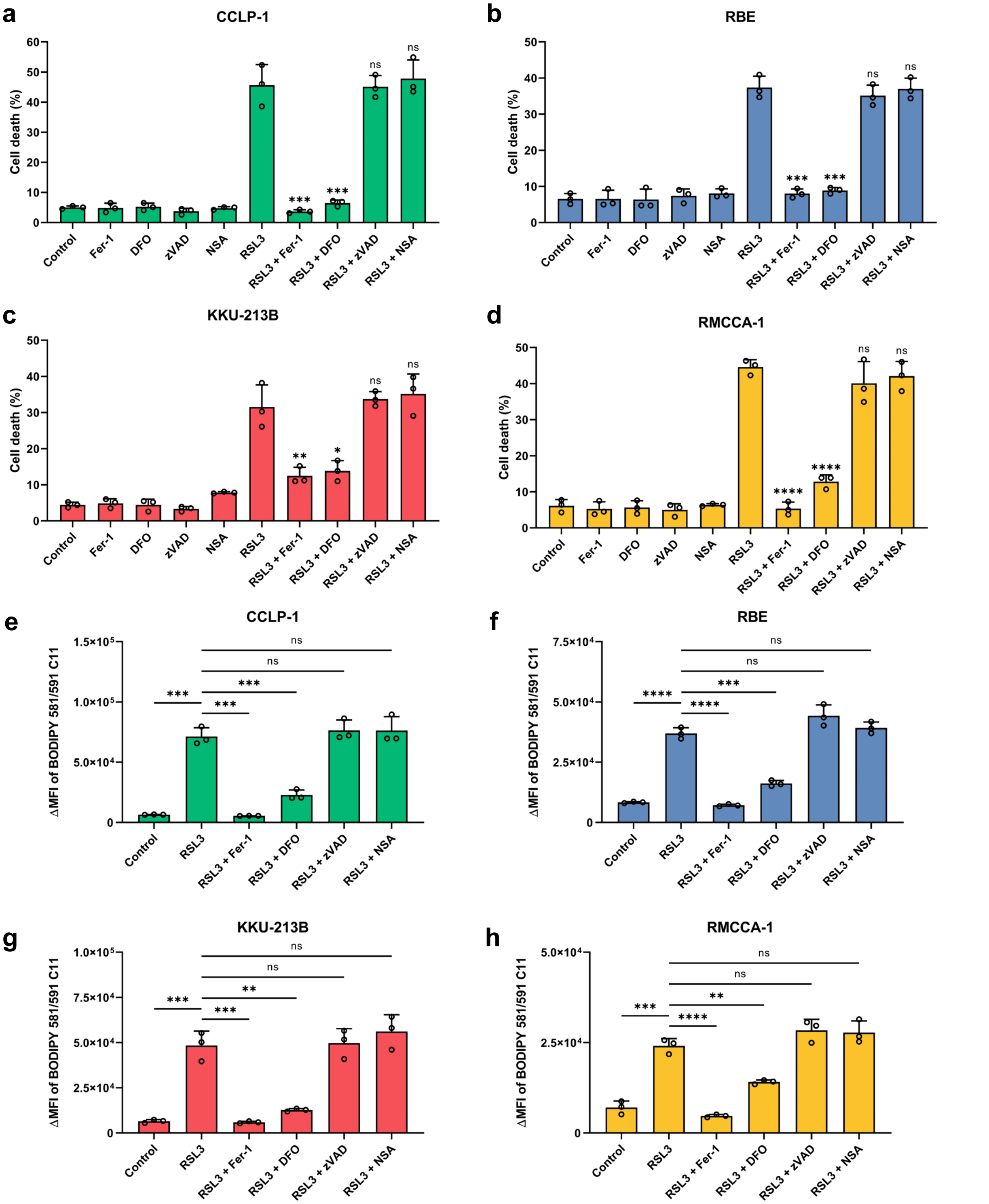


Supplementary Fig. 3 Confirmation of ferroptosis induction in CCA cell lines. (**a-d**) CCA cell lines were pretreated with 5 μM Fer-1, 10 μM DFO, 20 μM zVAD-fmk, or 1 μM NSA, then exposed for 48 h to RSL3 (0.05 μM for CCLP-1, 0.2 μM for RBE, 4 μM for KKU-213B, and 2 μM for RMCCA-1) and cell death was determined by flow cytometry using Annexin V/PI staining. (**e-h**) Lipid peroxidation in CCA cell lines pretreated with 5 μM Fer-1, 10 μM DFO, 20 μM zVAD-fmk, or 1 μM NSA, and exposed to RSL3 (0.05 μM for CCLP-1, 0.2 μM for RBE, 4 μM for KKU-213B, and 2 μM for RMCCA-1) for 6 h was by flow cytometry using BODIPY™ 581/591 C11 staining. All experiments were repeated independently three times. The data are shown as mean values ± S.D. and *p*-values were calculated using two-tailed unpaired Student's *t*-test. ns, not significant; *$p < 0.05$; **$p < 0.01$; ***$p < 0.001$; ****$p < 0.0001$.

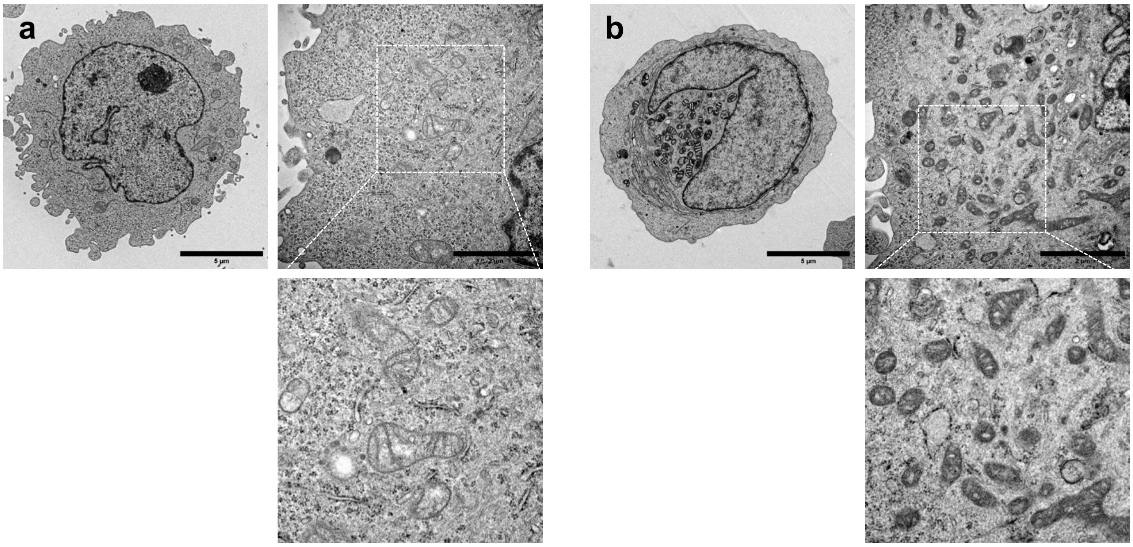


Supplementary Fig. 4 TEM micrographs of CCLP-1 cells treated with vehicle alone (**a**) or 25 nM RSL3 (**b**) for 8 h. Note increased cristae density and dilated ER in (**b**). Scale bars: 5 μm (overview) and 2 μm (close-up).

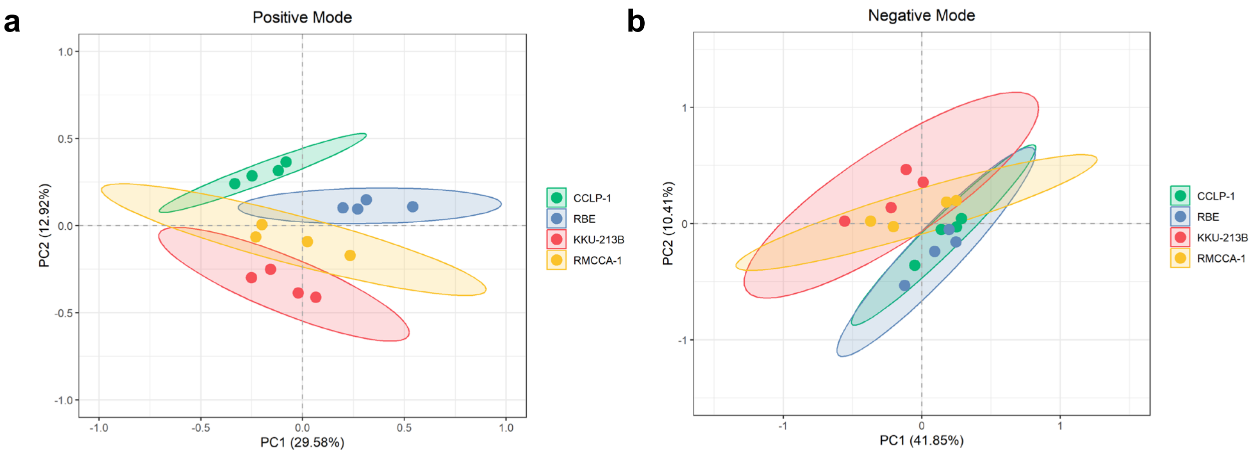


Supplementary Fig. 5 Principal component analysis. (**a**) PCA of lipidomics data obtained for the CCA cell lines in positive mode. (**b**) PCA of lipidomics for the CCA cell lines in negative mode.

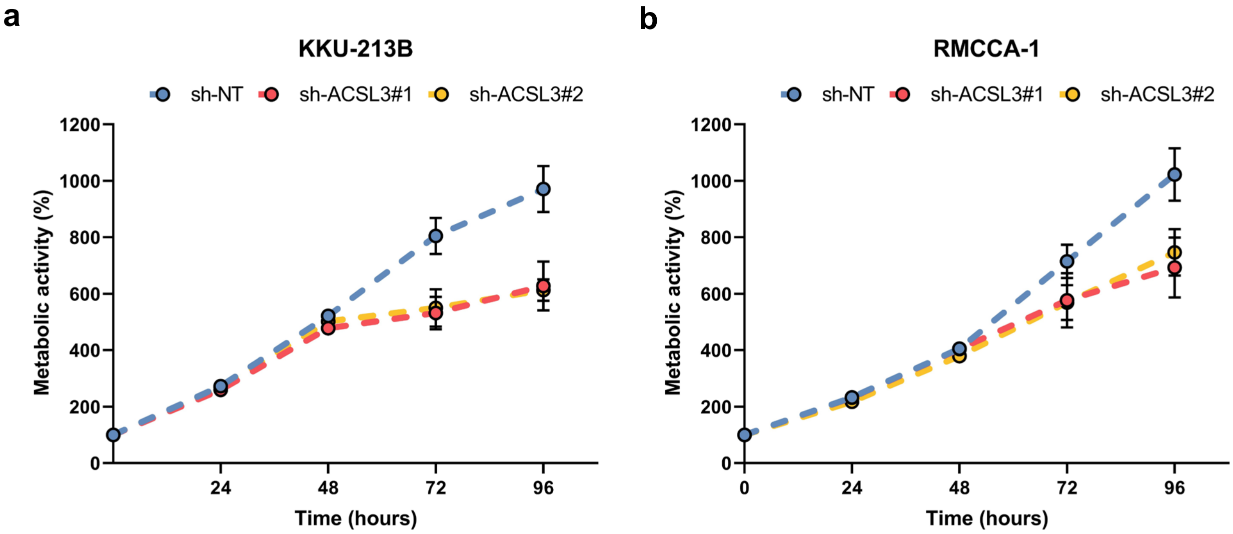


Supplementary Fig. 6 ACSL3 silencing compromises cell viability in CCA cells after 48 h of cell culture while no differences are seen up to 48 h. ACSL3-expressing and ACSL3-silenced KKU-213B cells (**a**) and RMCCA-1 cells (**b**) were cultured for 24, 48, 72, and 96 h and cell viability was determined using the MTT assay. All experiments were repeated independently three times. The data are shown as mean values ± S.D.

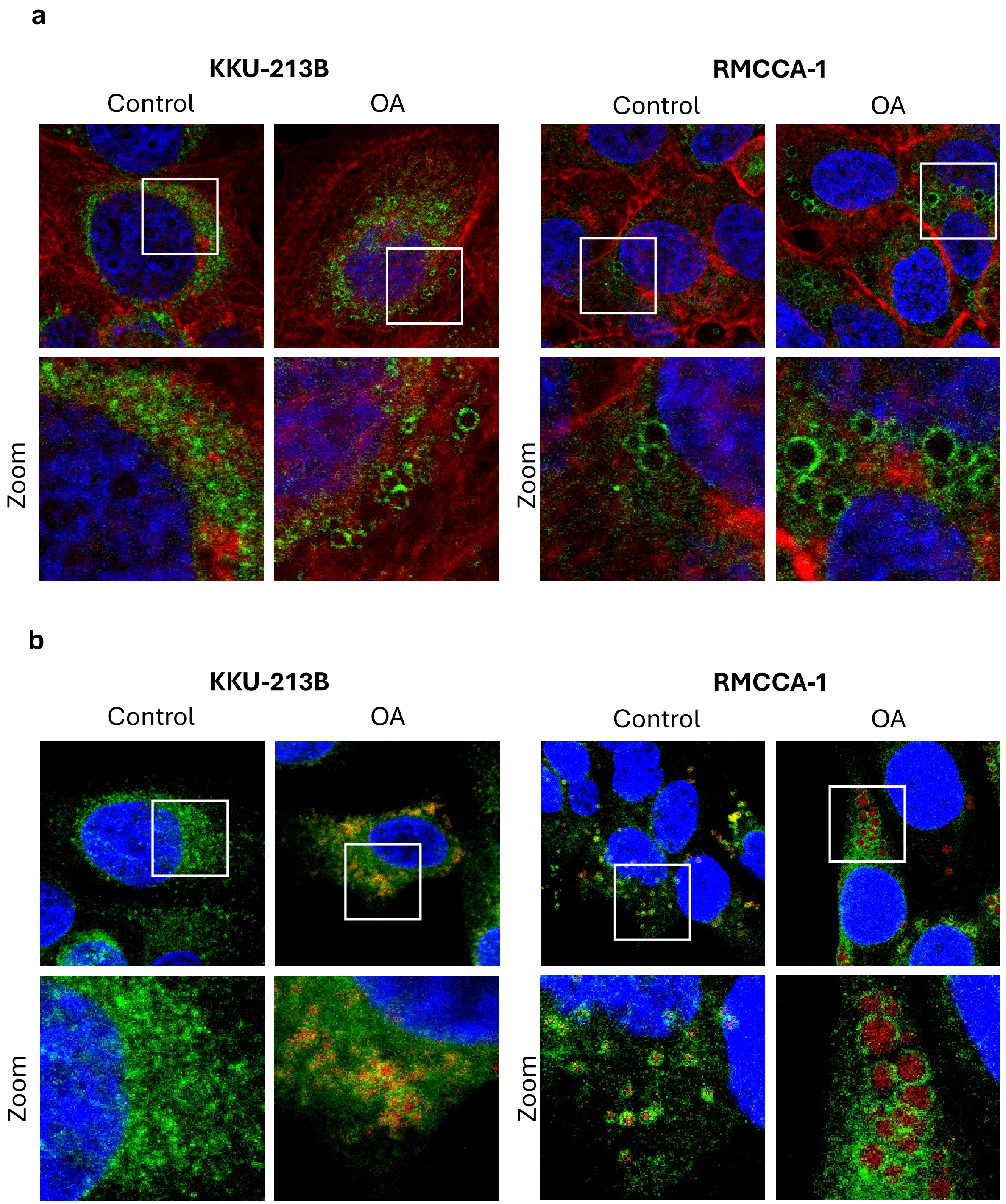


Supplementary Fig. 7 ACSL3 is localized to lipid droplets. The images shown here are a duplication of the images in Figure 7a, b with zoom-in images added showing the localization of ACSL3. (**a**) ACSL3 localization in KKU-213B and RMCCA-1 cells after exposure to vehicle control or OA (100 μM) for 24 h. Cells were stained with antibodies specific for ACSL3 (green) and counterstained with Hoechst 33342 (blue) (cell nuclei) and phalloidin-iFluor 647 (red). (**b**) Co-localization studies of ACSL3 and lipid droplets in KKU-213B and RMCCA-1 cells after exposure to vehicle control or OA (100 μM) for 24 h. Cells were stained with antibodies against ACSL3 (green) and cell nuclei were counterstained with Hoechst 33342 (blue), while lipid droplets were stained using Nile Red (red).

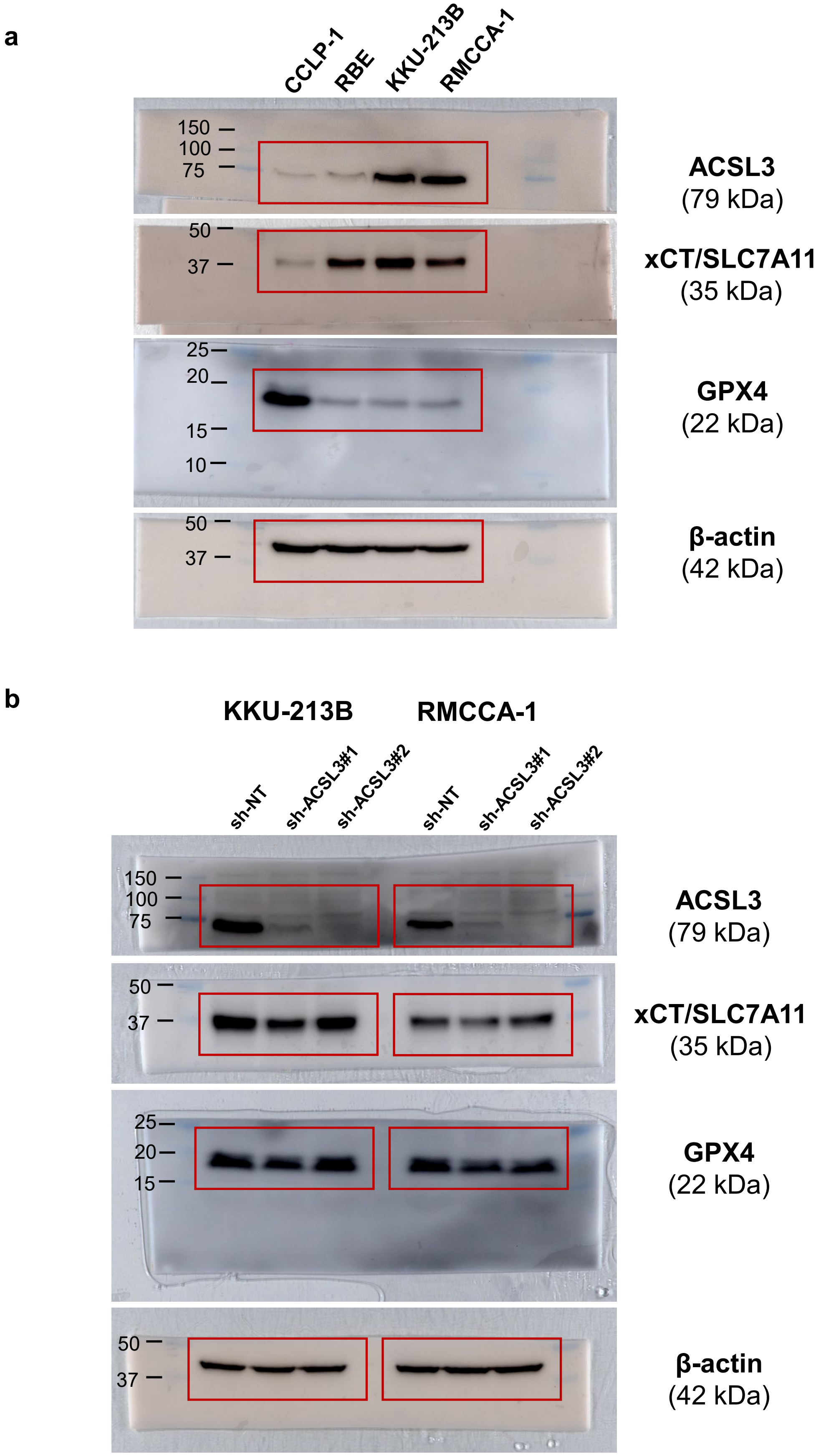


Supplementary Fig. 8 Original data for Western blot. The images shown here are uncropped and unprocessed Western blots corresponding to Figure 2b (**a**) and Figure 4a, b (**b**).
